# A Practical Study of Data Requirements for Self-Supervised Learning in Medical Image Analysis

**DOI:** 10.7759/cureus.98049

**Published:** 2025-11-28

**Authors:** Mitsuki Hommyo, Takumasa Tsuji, Shinobu Kumagai, Kenshiro Shiraishi, Jun'ichi Kotoku

**Affiliations:** 1 Graduate School of Medical Care and Technology, Teikyo University, Tokyo, JPN; 2 Development Division, Southwood Inc., Tokushima, JPN; 3 Central of Radiology, Teikyo University Hospital, Tokyo, JPN; 4 Department of Radiology, Teikyo University School of Medicine, Tokyo, JPN; 5 Health Data Science, Teikyo University, Tokyo, JPN

**Keywords:** low-data regime, moco, self-supervised learning, simclr, simsiam

## Abstract

Deep learning has become the mainstream approach for medical image analysis, but the availability of annotated datasets remains constrained in many clinical scenarios. Even when using such limited data, however, self-supervised learning (SSL) has yielded promising results. Nevertheless, comprehensive investigations into the number of images required for effective pretraining and fine-tuning in SSL frameworks remain inadequate. Especially lacking are such studies assessing factors such as class diversity, dataset scale, and task complexity. For this study, we evaluated the performance of widely adopted contrastive SSL models, particularly assessing their applicability in limited data settings by examining how the number of images used for pretraining and fine-tuning influences the accurate execution of binary classification tasks. Our findings indicate that among the three studied methods, Simple Siamese Representation Learning (SimSiam) achieved high accuracy based on only four training images. Achieving such performance would likely require at least 10,000 pretraining images. These findings offer practical insights into optimizing SSL-based pipelines for medical image analysis, particularly in scenarios involving rare diseases or severely scarce data.

## Introduction

Deep learning has revolutionized medical image analysis, supporting particularly important breakthroughs in classification, anomaly detection, and segmentation tasks [1,2]. However, a fundamental challenge persists: medical image acquisition remains prohibitively expensive, particularly for early stages of disease outbreaks, for which sample availability is severely constrained [3].

Transfer learning addresses such data scarcity by leveraging pretrained models, typically those trained using large-scale natural image datasets such as ImageNet [4]. Although ImageNet-pretrained models have demonstrated utility for medical applications [5], the considerably wide domain gap separating natural and medical images often limits their effectiveness when applied to specialized tasks such as lymph node metastasis detection [6]. Consequently, domain-specific pretraining on medical images has become fundamentally important, providing clear performance benefits [7] and additional support from increasingly available public datasets such as MedMNIST [8,9]. However, label-dependent pretraining approaches are hindered by three crucial limitations. First, medical image annotation is often costly [10]. Second, annotation errors can mislead model selection, as demonstrated by the 6% error rate of ImageNet test labels, which apparently led to ResNet18 outperforming ResNet50 [11]. Third, institutional and regional variations in imaging protocols entail a risk of teaching models to rely on spurious features rather than on clinically meaningful patterns [12].

Self-supervised learning (SSL) emerges as a promising solution that enables the learning of visual representations from unlabeled images without reliance on costly annotation. This approach theoretically addresses all three limitations: recent advances have demonstrated encouraging results in federated learning [13], anatomical modeling [14, 15], and patient metadata integration [16-19]. Comprehensive reviews [20, 21] have highlighted the growing adoption of SSL for medical imaging occurring along with improvements in architectures, training methodologies, and pretext task design.

Despite this progress, a fundamental issue related to the practical use of SSL in medical imaging remains: uncertainty about the number of images necessary for effective workflows. This issue manifests in two important dimensions. Given adequate pretraining, what is the minimum number of labeled images needed for effective fine-tuning while maintaining high accuracy? Current evidence provides limited guidance because of methodological inconsistencies, including variations in dataset classes, evaluation metrics, and lesion characteristics. Additionally, how does the number of pretraining images affect fine-tuning efficiency? Natural image studies show that reducing pretraining data from 1M to 250k images decreases top-1 accuracy by 2-4%. Moreover, further reduction to 50k engenders a greater than 10% accuracy loss [22]. Such large-scale medical image collections, therefore, remain impractical.

This study systematically investigates the effectiveness of SSL, with (1) elucidation of the relation between the fine-tuning dataset size and the ability of SSL models to maintain performance, (2) examination of how the pretraining dataset size influences this relation, and (3) analysis of how fine-tuning dataset characteristics differences affect the overall effectiveness of SSL. Through this study, we demonstrate the conditions under which SSL models can achieve competitive performance with only a few fine-tuned images.

## Materials and methods

This study used a two-stage experiment design to elucidate the relation between dataset size and SSL performance. The first stage used an SSL-pretrained model trained on a large dataset to examine the effects of varying fine-tuning dataset sizes on model performance. The second stage investigated how the number of pretraining images, in combination with more limited training image settings, affects performance.

**Figure 1:**
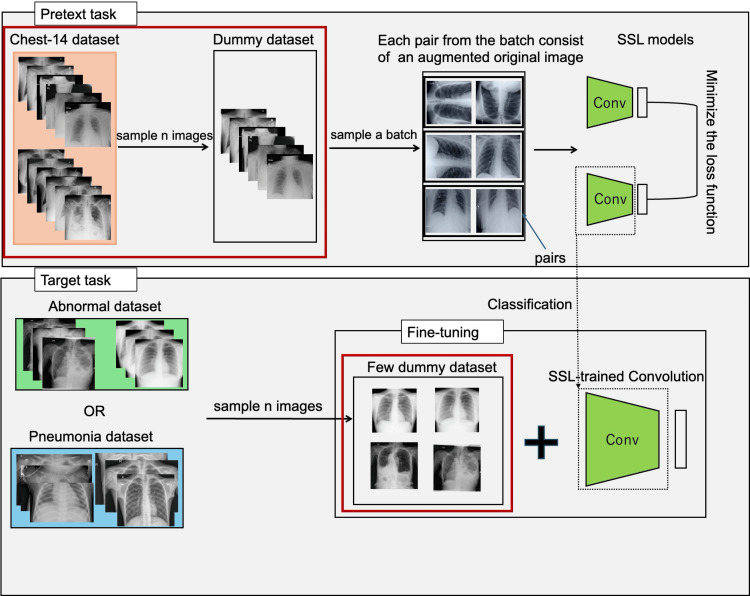
Overview of the training pipeline The red box in the target task section represents the first-stage analysis. The red boxes in the pretext task and target task sections represent the second-stage analysis. SSL: Self-supervised learning

The experiment framework comprised two sequential training phases: a pretext task for self-supervised pretraining and a target task for downstream classification fine-tuning. An overview of the complete training pipeline is depicted in Figure 1. Subsequent sections present details of the implemented SSL architectures, training protocols, and datasets used for this investigation.

Dataset description

Three datasets were used for this study: ChestX-ray14 [23], the abnormal dataset [24], and the pneumonia dataset [25]. The ChestX-ray14 dataset served exclusively for model pretraining, whereas the abnormal and pneumonia datasets were used for performance evaluation during fine-tuning experiments.

The ChestX-ray14 dataset comprises 112,120 chest X-ray images collected from 30,805 patients during 23 years (1992-2015). Each image is either labeled as “No Finding” or is annotated with one or more of 14 pathological conditions that had been extracted automatically from corresponding radiology reports using natural language processing. This dataset is publicly available for research purposes.

The abnormal dataset includes 3,032 chest X-ray images acquired at Teikyo University Hospital, consisting of 2,002 normal cases and 1,030 abnormal cases. Abnormal cases encompass diverse imaging conditions, including both standard posteroanterior views and portable X-ray examinations captured with patients in various positions such as standing, sitting, and supine. Ethical approval for use of this dataset was obtained from the Teikyo University Institutional Review Board (Approval No. 17-108-8).

The pneumonia dataset specifically examines pediatric chest X-ray images from patients aged 1-5 years, as obtained from Guangzhou Women and Children’s Medical Center. This publicly available dataset is partitioned into training and testing subsets, where the training set comprises 5,232 images, including 1,349 normal cases and 3,883 pneumonia cases (2,538 bacterial and 1,345 viral). The test set consists of 624 images, with 234 normal cases and 390 pneumonia cases (242 bacterial and 148 viral).

All datasets underwent consistent preprocessing, with images resized to 512 × 512 pixels from their original PNG format to ensure computational efficiency and consistency across experiments.

Contrastive self-supervised learning (SSL)

**Figure 2:**
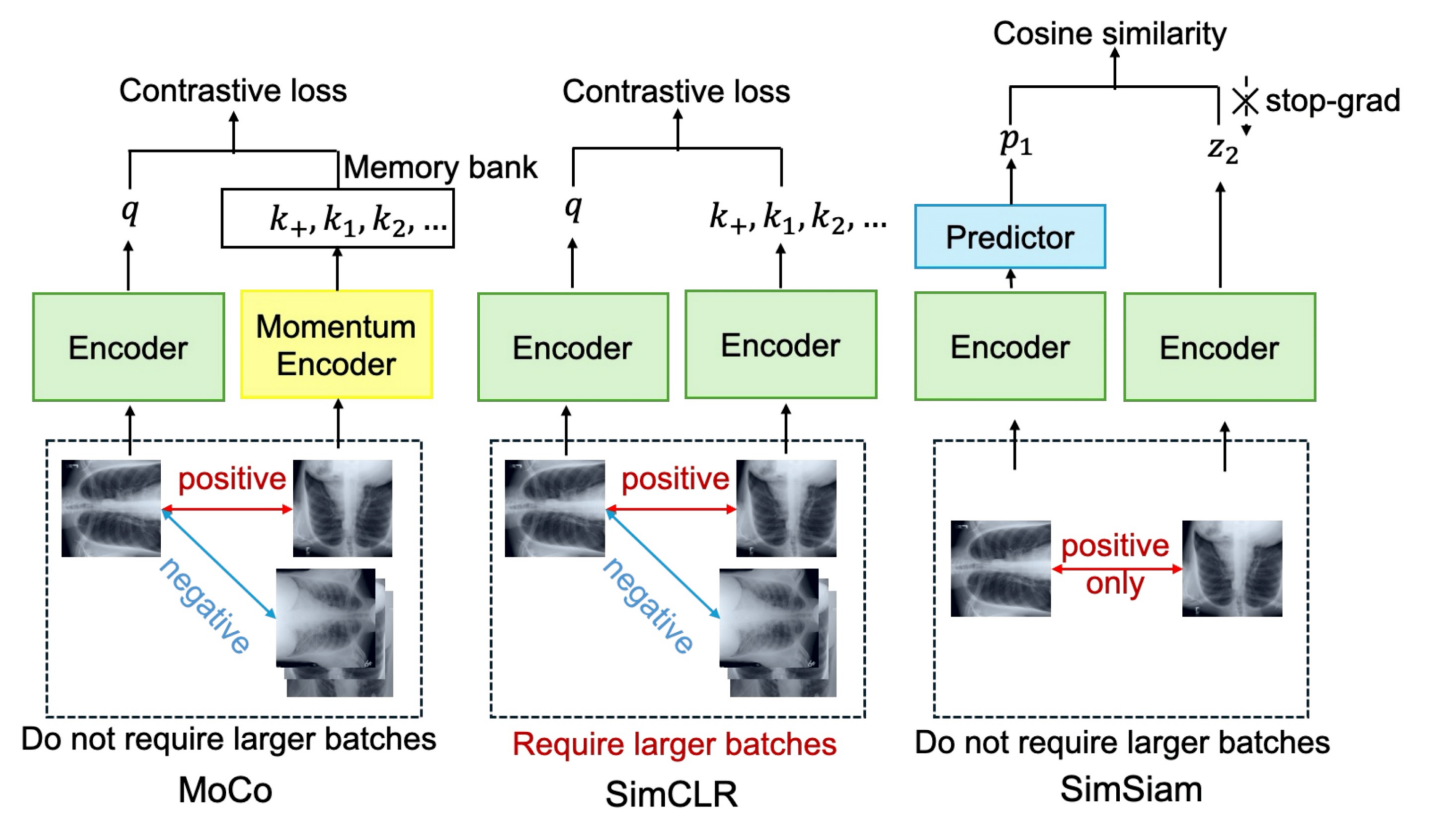
Contrastive SSL architectures The MoCo, SimCLR, and SimSiam model architectures are presented from left to right.

A salient benefit of SSL is that it enables the acquisition of visual representations from unlabeled image data without manual annotations. Among various SSL approaches, contrastive learning methods have demonstrated superior performance across classification and segmentation tasks [26]. This study implemented three established contrastive SSL architectures based on Siamese networks: MoCo, SimCLR, and SimSiam [27-31]. Figure 2 presents details of each method’s architectural components.

To address the computational constraints of contrastive learning, MoCo employs a dynamic dictionary mechanism [32]. Traditional contrastive learning benefits from the use of numerous negative pairs for each training iteration, but memory limitations restrict the practical batch size. MoCo circumvents this limitation by maintaining a queue-based dictionary of encoded features, updated via momentum, to ensure consistency across training iterations.

By virtue of its streamlined contrastive learning approach, SimCLR eliminates dependence on memory banks. Consequently, the framework achieves performance that is comparable to that of supervised methods by leveraging large batch sizes to provide sufficient negative pairs within each training batch, thereby obviating the need for external memory storage mechanisms.

By learning meaningful representations without negative pairs, SimSiam constitutes a paradigm shift. This approach eliminates the computational overhead associated with large batch sizes and memory banks required by earlier contrastive methods while maintaining competitive performance through asymmetric network design and stop-gradient operations.

Dataset preparation for contrastive self-supervised learning (SSL)

Contrastive SSL training requires the systematic construction of positive and negative pairs to facilitate representation learning. Positive pairs generated by application of distinct data augmentation transformations to identical source images create correlated views that preserve semantic content while introducing visual variation. Negative pairs were generated by application of data augmentation transformations to different source images, thereby producing views that are assumed to be semantically dissimilar.

The inherent characteristics of medical images present distinctive challenges for contrastive learning frameworks. Medical images exhibit a marked degree of inter-sample similarity because of consistent anatomical positioning and subtle inter-class visual distinctions. This similarity increases the risk of constructing negative pairs that are not genuinely dissimilar, possibly inducing dimensional collapse [33]: a phenomenon that constrains the model's ability to learn diverse and discriminative representations [34].

To mitigate these challenges while preserving task-relevant anatomical information, we implemented a carefully balanced augmentation protocol. Rotation transformations were incorporated to disrupt anatomical position consistency and to enhance negative pair dissimilarity, although rotation has been reported to degrade performance in certain tasks [35]. The final augmentation pipeline comprised the following: color jittering with conservative intensity parameters to maintain tissue contrast characteristics, random cropping with minimum retention of 50% of the original image area to preserve anatomical context, horizontal flipping applied with 50% probability to introduce spatial variation, and discrete rotation (90°, 180°, 270°) applied with 50% probability to reduce positional similarity. This augmentation strategy follows the guidelines of the Lightly SSL framework tutorial to ensure robust contrastive learning while adapting to the characteristics of medical images.

Training pipeline

The training process comprised two sequential phases. The first phase involved a pretext task for SSL-pretraining, followed by a target task for supervised fine-tuning. Given the limited training data availability, ResNet-18 [36] was selected as the backbone architecture because of its lightweight design. It demonstrated some effectiveness for small datasets because models with fewer parameters have been shown to achieve better performance in data-limited scenarios [37].

For the pretext task, the contrastive SSL architectures and associated training parameters were configured according to recommendations from Lightly, a comprehensive framework for SSL implementations. Contrastive SSL models were trained using a Stochastic Gradient Descent (SGD) optimizer [38] with a batch size of 16 across 300 epochs. Loss functions differed according to each SSL method. For both MoCo and SimCLR implementations, NT-Xent loss [30] was applied, whereas SimSiam employed the original loss function proposed in its foundational work. Table 1 presents details of additional hyperparameters.

**Table 1:** Hyperparameters for SSL The memory bank represents the size of the dictionary used for MoCo. Temperature denotes the temperature hyperparameter used in the contrastive loss function.

Architecture	Memory Bank	Learning Rate	Temperature	Scheduler
MoCo	4096	6.0e-2	0.1	Cosine Annealing
SimCLR	-	6.0e-2	0.5	Cosine Annealing
SimSiam	-	3.1e-4	-	-

For the target task, the downstream dataset was partitioned into 80% for training and 20% for validation, with the pretrained representations subsequently fine-tuned on the training portion. For comparative analysis, we evaluated SSL-pretrained models alongside ImageNet-pretrained and randomly initialized baselines. Fine-tuning used cross-entropy loss with Adam optimizer using a batch size of 64, an initial learning rate of 1e-4 with a decay factor of 10 every 7 epochs, and early stopping after 15 epochs without validation improvement, with a maximum of 50 training epochs. However, for the second-stage settings with few images, we instead used 20 validation images, set the initial learning rate to 1e-4 with a decay factor of 10 every 15 epochs, and omitted early stopping. The experiments were conducted using Python 3.8 and three GPUs (RTX A6000; NVIDIA Corp.), each equipped with 49,140 MiB of memory.

Performance evaluation

The relation between dataset size and SSL performance was assessed through comprehensive qualitative and quantitative evaluations. Qualitative analysis employed t-SNE visualization to evaluate the representation quality of pretrained features [39,40]. Quantitative evaluation used classification accuracy as the primary performance metric.

Two experiment paradigms were implemented to investigate dataset size effects. First, we examined fine-tuning performance variations using SSL models pretrained on 112,120 images. Feature quality was assessed through t-SNE visualization of extracted representations from the pneumonia dataset across all three SSL methods. Performance was then quantified by reducing fine-tuning dataset sizes systematically to approximately 2,000, 1,000, 500, 250, 100, 50, and 20 images, measuring the classification accuracy using standardized test sets. The abnormal dataset test set comprised 100 normal and 100 abnormal chest X-rays, whereas the pneumonia dataset used the original predefined test partition. To ensure statistical robustness, 10 random resampling iterations were performed for each fine-tuning configuration.

Second, while examining performance under more limited training image settings, we investigated how the number of pretraining images influences performance in such scenarios. Using the SSL method, which demonstrated superior fine-tuning performance, pretraining datasets were randomly reduced systematically to 112,120, 10,000, 1,000, and 100 images. Moreover, fine-tuning was examined further, down to the one-shot setting, with performance evaluated using identical methodological protocols. In addition, area under the curve (AUC) was computed to provide a more comprehensive evaluation of classification performance.

## Results

SSL-model Performance

**Figure 3:**
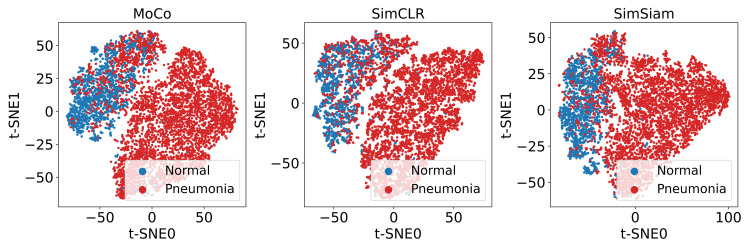
t-SNE visualization for evaluating SSL-pretraining effectiveness The pneumonia dataset was used, with blue representing normal cases and red representing pneumonia cases. The visualized feature vectors were extracted from the images and were projected into two dimensions. From left to right, the results correspond respectively to models pretrained using MoCo, SimCLR, and SimSiam.

We first used t-SNE visualization to assess the quality of learned feature representations obtained using the three SSL methods. The t-SNE plots revealed clear separation between pneumonia and normal cases for features extracted using MoCo, SimCLR, and SimSiam, indicating successful pretraining across all methods. The t-SNE visualization of extracted image features is presented in Figure 3.

**Figure 4:**
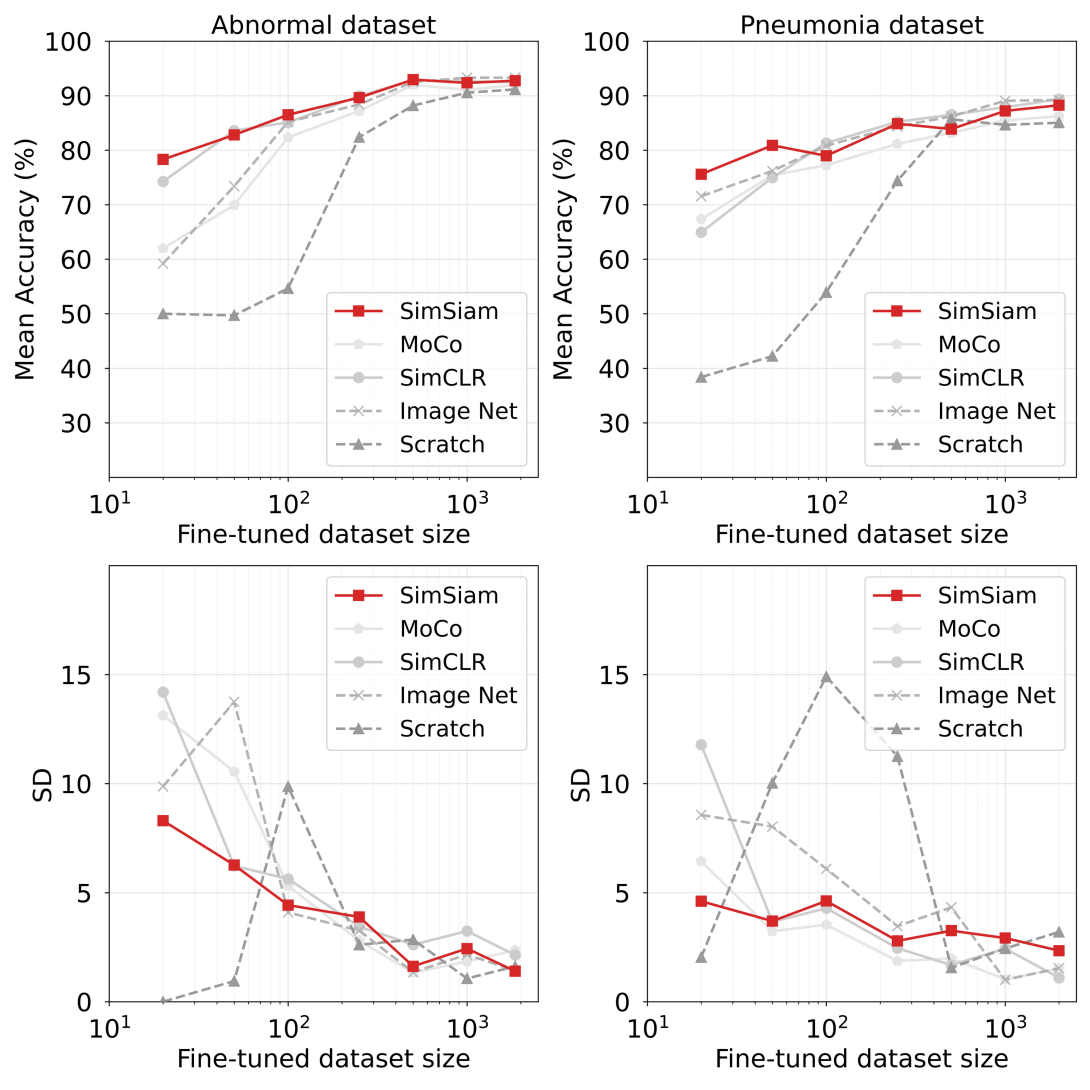
Classification performance as a function of training dataset size for different experiment conditions Upper panels display the mean classification accuracy averaged over 10 independent trials, whereas lower panels show the corresponding standard deviations, indicating performance variation and model stability.

Generally speaking, SSL-pretrained models outperformed the ImageNet-pretrained baseline for both datasets, with the most pronounced performance gains achieved in low-data scenarios. Details of the accuracy results are presented in Figure 4. With the abnormal dataset, SimSiam demonstrated the strongest performance in the 20-image fine-tuning condition, markedly outperforming both the ImageNet baseline and other SSL methods. When the number of fine-tuning images was increased to 50, both SimSiam and SimCLR achieved performance exceeding that of ImageNet-pretraining. However, with 100 or more fine-tuning images, the performance gap separating SSL methods and ImageNet-pretraining became negligible, suggesting that the advantage of SSL-pretraining is most evident in data-scarce scenarios.

Similar trends were observed for the pneumonia dataset, but with smaller performance margins. SimSiam consistently achieved superior performance across different fine-tuning dataset sizes, with the most notable improvements observed for under 50-image dataset size conditions. The performance differences between SSL methods and ImageNet-pretraining diminished as the number of fine-tuning images increased.

Model decision visualization with grad-CAM

**Figure 5:**
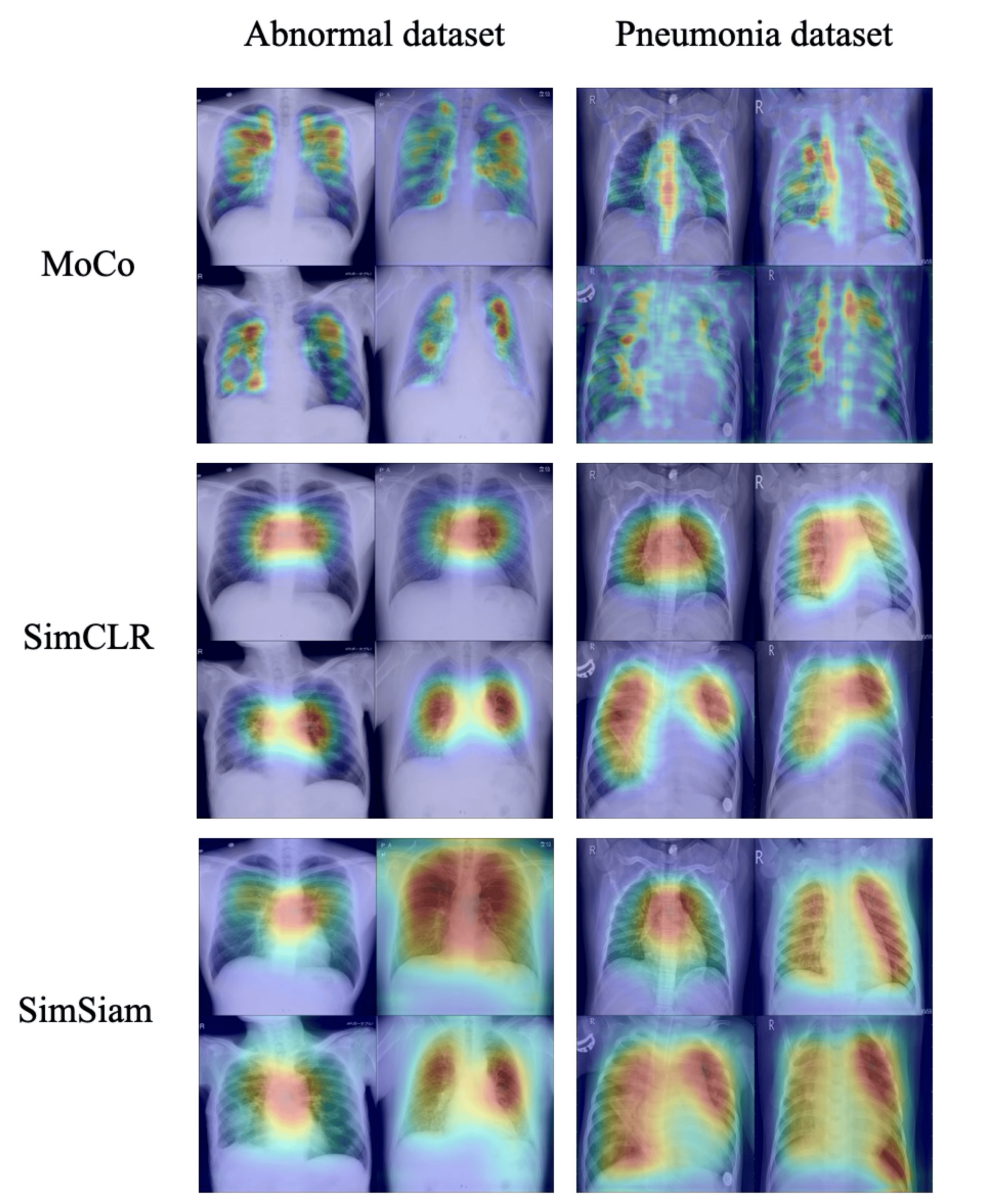
Grad-CAM heatmaps for models fine-tuned with 20 images For each model, the top row shows normal chest X-rays, whereas the bottom row shows abnormal (pneumonia) cases.

To understand the superior performance of SSL-pretrained models in low-data regimes, we used Grad-CAM visualizations to analyze their decision-making processes. Attention heatmaps revealed distinct focusing patterns among the three SSL methods, as presented in the corresponding Figure 5.

Although MoCo predominantly attended to structural elements such as the ribs and spine, SimCLR concentrated on the heart and surrounding lung regions. Both models exhibited consistent attention patterns across different inputs. By contrast, SimSiam demonstrated more precise attention to lung and heart structures, which are crucially important for classification tasks. It is noteworthy that SimSiam showed input-dependent attention variation, suggesting a more sophisticated understanding of diagnostically relevant features, which might explain its superior classification performance.

Effects of pretraining dataset size

**Figure 6:**
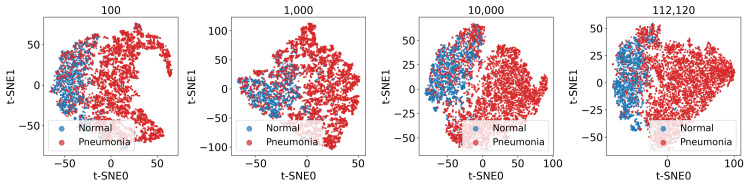
t-SNE visualization of visual representations learned by SimSiam with different quantities of pretraining images The pneumonia dataset was used, where blue represents normal cases, and red represents pneumonia cases. From left to right, the results correspond to SimSiam models pretrained on 100, 1,000, 10,000, and 112,120 chest X-ray images.

Given SimSiam’s superior performance with limited fine-tuning data, we investigated how the number of pretraining images, in combination with more limited training image settings, affects performance. As depicted in Figure 6, t-SNE visualization revealed that SimSiam separated normal and pneumonia cases irrespective of the pretraining dataset size. It is particularly interesting that the feature distribution from the model pretrained on 10,000 images closely resembled that of the model pretrained on the full dataset (112,120 images), suggesting efficient feature learning, even with limited pretraining data.

**Figure 7:**
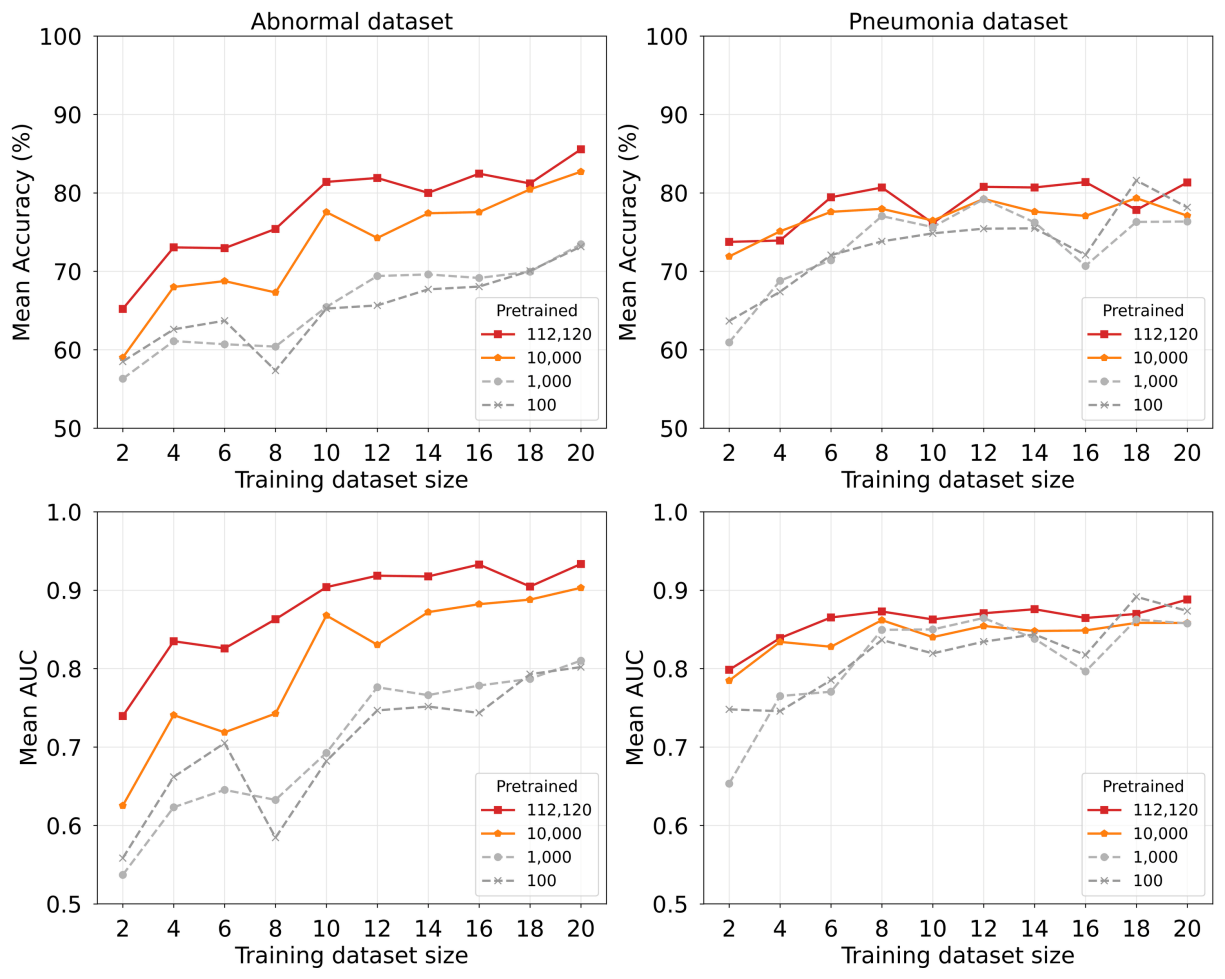
Classification accuracy and AUC as a function of pretraining dataset size across limited training image settings Each line represents a different pretrained dataset size, as presented in the legend. The figure displays the mean classification accuracy and AUC averaged over 10 independent trials.

Classification results obtained across different pretraining dataset sizes and more limited training image settings are presented in Figure 7. For both datasets, the model pretrained on the full dataset achieved average accuracy exceeding 70%, even after using only four training images, whereas the models pretrained on 100 and 1,000 images were found to have lower and similar performance. In contrast, dataset-specific differences were also observed. With the abnormal dataset, increasing pretraining data from 10,000 to the full dataset improved accuracy by approximately 5%, particularly when training with smaller amounts of data. However, the benefit of pretraining with a full dataset was not remarkable, corresponding to up to 10 images. AUC values showed a similar trend to that shown by accuracy, indicating consistent performance behavior among different pretraining conditions.

It is particularly interesting that the pneumonia dataset was found to have different sensitivity to the pretraining dataset size. Both pretraining conditions achieved comparable performance across different training dataset sizes, with only modest differences observed. This outcome suggests that the pneumonia classification task might be less dependent on extensive pretraining data, possibly because of the more distinct visual features it involves compared to the abnormal detection task.

## Discussion

We evaluated the SSL effectiveness for small-scale medical image analysis and assessed how the pretraining dataset size, the fine-tuning dataset size, and the classification task characteristics affect model performance. Our results demonstrate that SSL-pretraining mitigates performance degradation effectively when only four training images are available. It is particularly noteworthy that the SimSiam model pretrained on 10,000 images also achieved high average accuracy, demonstrating its potential to maintain high classification performance even in data-scarce environments.

Superior performance of SimSiam and insights from visual representation learning

SimSiam outperformed other SSL methods consistently across the experiments described herein. Grad-CAM visualizations provided crucially important insights into why SimSiam achieves superior performance, revealing fundamental differences in how SSL approaches the processing of medical images. When fine-tuned with only 20 images, SimSiam demonstrated superior capability for adaptive and specific examination of different anatomical regions depending on the classification label, thereby capturing clinically relevant structures such as the heart and lungs effectively. By contrast, MoCo and SimCLR exhibited consistent attention patterns with minimal variation across different labels. This adaptive attention mechanism of SimSiam represents an important benefit because it suggests that the model learns to identify label-specific features rather than relying on fixed visual patterns.

This superior performance can be attributed to SimSiam’s unique architecture, which avoids negative pairs by relying on a stop-gradient mechanism to prevent representational collapse. Unlike contrastive methods such as MoCo and SimCLR, SimSiam requires no carefully constructed negative samples. Obviation of those samples might be particularly beneficial for medical imaging, for which anatomically similar regions can confound contrastive learning objectives.

Our findings suggest that contrastive learning methods might be inherently less suitable for single-modality medical images because of challenges posed by constructing effective negative pairs from anatomically similar structures. MoCo and SimCLR consistently emphasized regions that might not optimally capture the relevant anatomical structures, potentially because of difficulty in learning discriminative features when negative pairs comprise similar anatomical structures. Earlier research has indicated that such negative pairs can hinder effective representation learning and can engender training instability in contrastive frameworks [34].

Each SSL method exhibited distinct emphasis on behaviors that reflect its underlying algorithmic approaches. Particularly, MoCo tended to concentrate on regions with high positional variability, such as ribs, probably because of its momentum-based feature dictionary, which becomes sensitive to spatially variable elements across the stored representations. The momentum mechanism might cause the model to prioritize features that show the greatest variation across the maintained dictionary of past representations.

Actually, SimCLR exhibited preferences for heart and lung size variations, reflecting its batch-based learning approach, which emphasizes inter-sample differences within each training batch. This preferential behavior suggests that SimCLR becomes particularly sensitive to features that exhibit the most pronounced variation among samples processed simultaneously during training.

By contrast, SimSiam identified clinically relevant anatomical landmarks and pathological regions consistently, suggesting more effective feature learning for medical image analysis. The absence of negative pairs in SimSiam’s architecture apparently allows the model to examine intrinsically important medical features specifically, rather than being distracted by the comparative relations that drive contrastive learning methods.

Optimization of pretraining data requirements

Our investigation into pretraining data scaling revealed task-dependent saturation patterns, providing insights into the complexity of various medical image classification difficulties. Analysis of dataset characteristics revealed behavioral patterns that depend on the classification task characteristics, particularly in terms of how image features affect model performance.

For the abnormal dataset, SimSiam’s performance continued improving even with 112,120 pretraining images, indicating that more complex classification tasks benefit from extensive pretraining data. The abnormal label encompasses diverse pathological conditions without a consistent visual pattern, requiring that the model learn a broader range of visual representations to achieve optimal performance.

By contrast, the pneumonia dataset showed performance saturation at approximately 10,000 pretraining images, suggesting that tasks with more consistent discriminative features require less extensive pretraining to achieve optimal performance. The uniform presentation of pneumonia allows the model to learn necessary distinguishing characteristics rapidly without requiring exposure to vast amounts of training data.

Importantly, the results of this study suggest that when the pretraining images are fewer than 1,000, the benefits of SimSiam pretraining might not be fully realized. In scenarios with limited training data, having approximately 10,000 pretraining images appears to be advantageous for achieving higher classification performance. Furthermore, incorporating SSL approaches that leverage patient metadata [16-19] might reduce the number of required pretraining images while still attaining comparable performance improvements.

Limitations

This study has several important limitations, which must be considered when interpreting these results and planning future research. The small batch sizes used for these experiments might have particularly disadvantaged SimCLR, which typically benefits from larger batch sizes for effective contrastive learning. This technical constraint arose from the computational requirements of processing high-resolution medical images. Those requirements restricted the number of images that could be processed simultaneously on the available GPU hardware. Future studies with access to more powerful computational resources might yield different comparative outcomes.

Our data augmentation strategies might not have been fully optimized for medical imaging applications. Advanced augmentation methods, such as mask-based approaches or domain-specific transformations designed for medical images, were not used for this study. The implementation of more sophisticated augmentation techniques might reduce pretraining data requirements while improving overall model performance across all SSL methods.

The scope of this investigation was limited to chest X-ray images, which were selected for their complexity, diverse pathological patterns, and widespread clinical use. Although these characteristics make chest X-rays broadly representative of medical imaging challenges, generalization to other medical imaging modalities requires further investigation. Different imaging techniques, such as CT scans, MRI, ultrasound, and histopathological images, because of their unique visual characteristics and diagnostic requirements, might elicit different responses to SSL methods.

Despite these limitations, the findings presented herein provide valuable insights into the application of SSL methods for medical image analysis. Moreover, this study provides practical guidance for implementing these techniques in clinical settings with limited annotated data, helping to minimize the risk of AI model failure caused by insufficient training images. Our demonstration of the superior performance of SimSiam for medical imaging applications represents an important step toward developing more effective AI tools for healthcare applications.

## Conclusions

This study investigated strategies for maintaining CNN performance in limited data situations, which are characteristic of rare diseases and specialized medical examinations. Our results demonstrate that SimSiam SSL provides considerable performance gains for binary classification tasks when the training datasets are severely limited to four images. Although the optimal pretraining dataset size varies according to task complexity, the findings reported herein suggest that approximately 10,000 images represent a practical minimum threshold for effective SimSiam pretraining.

These results provide actionable guidance for developing robust CNN architectures in medical domains that have limited data. The demonstrated efficacy of SSL extends beyond rare disease classification to more complex tasks, for which subtle imaging features are often difficult to interpret. In such challenging scenarios, SSL frameworks such as SimSiam not only enhance predictive accuracy: they also provide valuable insights into model performance limitations. Our work provides a foundation for deploying deep learning solutions in underserved medical specialties, potentially accelerating diagnostic capabilities in areas where traditional supervised learning approaches remain impractical because of data limitations.
